# Living myocardial slices retain patient-specific features: Insights into etiology and therapeutic history

**DOI:** 10.1016/j.jhlto.2025.100345

**Published:** 2025-07-14

**Authors:** Jort S.A. van der Geest, Willem B. van Ham, Ernest Diez Benavente, Mohsin El Amrani, M. Mostafa Mokhles, Marish I.F.J. Oerlemans, Pieter A. Doevendans, Teun P. de Boer, Joost P.G. Sluijter, Linda W. van Laake, Vasco Sampaio-Pinto

**Affiliations:** a Department of Cardiology and Experimental Cardiology Laboratory, Division of Heart & Lungs, University Medical Centre Utrecht, Utrecht, the Netherlands; b Regenerative Medicine Centre Utrecht, Circulatory Health Research Center, University Utrecht, University Medical Centre Utrecht, Utrecht, the Netherlands; c Department of Medical Physiology, Division of Heart & Lungs, University Medical Centre Utrecht, Utrecht, the Netherlands; d Department of Clinical Pharmacy, Division Laboratory, Pharmacy and Biomedical Genetics, University Medical Centre Utrecht, Utrecht, the Netherlands; e Department of Cardiothoracic Surgery, University Medical Centre Utrecht, Utrecht, the Netherlands; f Transplantation Center University Medical Centre Utrecht, Utrecht, the Netherlands; g Netherlands Heart Institute (NLHI), Utrecht, the Netherlands; h Central Military Hospital (CMH), Utrecht, the Netherlands

**Keywords:** living myocardial slices, translational research, patient-specific model, heart transplant, LVAD, amiodarone

## Abstract

**Background:**

Living myocardial slices (LMS) are an emerging translational *ex vivo* model for studying myocardial function, disease mechanisms, and therapeutics. However, the extent to which *ex vivo* findings correlate with clinical characteristics is unknown. This study aimed to evaluate whether LMS retain patient-specific functional and pathological characteristics, reflecting diverse etiologies, pharmacological regimens, and clinical interventions.

**Methods:**

300-µm-thick LMS were prepared from myocardial biopsies of end-stage heart failure patients (*N* = 12, *n* = 138). Functional assessment of freshly prepared LMS included refractory period, stimulation threshold, force-frequency relationship, post pause potentiation, contractile force, alongside simultaneous optical recordings of calcium transients and action potentials. Variability and grouping analyses were conducted to identify features linked to patient-specific parameters, such as etiology and therapeutic history, including prior left ventricular assist device (LVAD) implantation and amiodarone usage.

**Results:**

LMS exhibited lower intrapatient variability (LMS from the same patient) compared to interpatient variability (LMS from different patients), confirming their ability to retain patient-specific functional properties. LMS from LVAD-treated patients exhibited reduced intrapatient variability and reduced diastolic tension, correlated with lower N-terminal pro-B-type natriuretic peptide levels. Stratification by etiology revealed distinct functional characteristics, including enhanced contractile force in titin-mutant LMS and a positive force-frequency relationship in ischemic cardiomyopathy-derived LMS. LMS derived from amiodarone-treated patients demonstrated prolonged action potential duration, reduced excitability at higher pacing frequencies, and enhanced post pause potentiation, reflecting the drug’s established pharmacological effects.

**Conclusions:**

LMS effectively capture distinct functional parameters associated with patient-specific features. These findings establish LMS as a valuable translational platform for personalized cardiac research, therapeutic testing, and precision medicine.

## Background

Advanced heart failure (HF) is a complex syndrome and a collective term for the inability of the heart to pump blood effectively and meet the body’s demand for oxygen and nutrients under normal physiological demands. While oxygen delivery may remain sufficient under elevated filling pressures, advanced HF denotes a stage where compensatory mechanisms are exhausted and tissue perfusion becomes inadequate despite such adaptations. Heterogeneity arises from the diverse underlying causes of the disease and is further shaped by the variety of pharmacological and clinical treatments employed in its management. While current HF treatments have significantly improved symptom relief and slowed disease progression, they do not cure the underlying disease, highlighting the need for innovative, disease-modifying therapies.^1^

The development of transformative therapies is hampered by the limited translatability of existing preclinical models. Animal models and *in vitro* systems often fail to replicate the complexity of human cardiac pathophysiology due to interspecies differences,^2^ immaturity of stem cell-based models,^3^ and their inability to mimic the multicellular organization, extracellular matrix, and biomechanical dynamics of the human myocardium. Furthermore, these models lack the capacity to reflect the cumulative impact of years of pathological remodeling observed in human HF. Therefore, more reliable and physiologically relevant research platforms are needed to enhance our understanding of cardiovascular diseases and accelerate the development of innovative, targeted therapies.

Living myocardial slices (LMS), 100 to 400 µm slices of myocardial tissue, retain the native cardiac architecture, preserving multicellular and extracellular matrix composition. Importantly, LMS are derived directly from patient hearts, inherently carrying the biological and mechanical signatures of years of pathological remodeling. While this unique feature offers the potential to retain and reflect patient-specific characteristics, such as the underlying etiology, disease severity, and response to therapeutic regimens, it could also introduce confounding factors. Retaining patient-specific features in an *ex vivo* model is critical for accurately representing clinical heterogeneity and advancing precision medicine, but systematic evaluation is required to understand the extent to which these features are preserved and how they influence experimental outcomes.

In this study, we employed LMS to assess their ability to retain patient-specific functional and pathological characteristics. By integrating acute functional assessments with simultaneous optical measurements of calcium and action potential dynamics in LMS, we explored their potential as a translational platform for advancing disease modeling, therapeutic testing, and precision medicine. Leveraging a diverse cohort of patients with varied etiologies, pharmacological regimens, and mechanical unloading histories, we aimed to evaluate whether LMS consistently capture functional distinctions associated with these clinical subgroups. The even distribution of patients across subgroups provides an opportunity to further assess the robustness of LMS in reflecting diverse patient-specific characteristics.

## Methods

### Study approval

The study was approved by the local medical ethics review board, and all patients provided written informed consent for the myocardial biopsies under UCC-UNRAVEL #12-387.^4^

### LMS preparation

Our study examined biopsies of 12 consecutive surgical procedures. LMS were generated from fresh myocardial tissue biopsies from the left ventricle of patients with end-stage HF. These biopsies were obtained from explanted hearts after transplantation, immediate postmortem examination, or apical biopsy during left ventricular assist device (LVAD) implantation. All samples were derived from individuals with advanced cardiac dysfunction, refractory to pharmacological therapies, requiring surgical intervention. The postmortem sample was collected from a donor with terminal HF on LVAD destination therapy, who had provided consent for scientific use, and the tissue was acquired during obduction promptly following death. To maximize viability and minimize tissue damage, all myocardial samples were collected quickly, within a warm ischemia interval not exceeding 30 minutes. Importantly, LMS produced from the sample collected postmortem had a similar viability and performance compared to those produced from samples collected intraoperatively, thus indicating equivalent storage efficacy. The myocardial biopsies were transported in an ice-cold modified Tyrode's solution (30 mM 2,3-butanedione monoxime, 0.9 mM CaCl_2_, 10 mM glucose, 10 mM 4-(2-hydroxyethyl)-1-piperazineethanesulfonic acid (HEPES), 9 mM KCl, 1 mM MgCl_2_, and 140 mM NaCl at a pH of 7.4). The baseline characteristics of the patients from whom the LMS were derived are presented in Table 1, while Table 2 details the distribution of stratifications used in this study. 300-µm-thick LMS were generated using a high-precision vibratome (z axis error <1.0 µm, frequency of 80 Hz, 2 mm amplitude, and an advance speed of 0.03 mm/s) (7000smz-2, Campden Instruments Ltd, Gillingham, UK), as described before.^5^ The collected myocardial biopsy was mounted with the epicardial side facing down. The slices were trimmed and secured to triangles cut from a 0.1-mm-thick polyester sheet perpendicular to the myofibril direction, using histoacryl glue (B. Braun, Melsungen AG, Germany). Generated LMS were continuously electrically paced (3 ms biphasic 50 mA pulses, at 0.5 Hz), stretched to a physiological level of 19.6%, and rocked at 60 rpm in biomimetic culture chambers (InVitroSys, München, Germany). The LMS were placed in medium 199 supplemented with 1% Insulin-Transferrin-Selenium (ITS), 0.2% primocin, 2.15 nM triiodothyronine (T3), 100 nM dexamethasone, 4 nM adrenaline, 4 nM noradrenaline, 50 μM 2-mercaptoethanol, and 20 μg/mL ascorbic acid (all from Sigma-Aldrich, Amsterdam, Netherlands). Acute functional characterization of a minimum of 5 slices per patient was performed using a custom-made stimulation protocol to determine the refractory period, stimulation threshold, force-frequency relationship, post pause potentiation, and contractile force. LabChart 8 Reader application from AD Instruments was used to conduct blinded data analysis.

**Table 1:** Baseline Characteristics of Patients’ Biopsies for LMS Generation

N	12
Age (years, median [interquartile range- IQR])	52 [51, 62]
Sex = M, *n* (%)	10 (83%)
Prior LVAD = Y, *n* (%)	9 (75%)
LVAD support duration (days, median [IQR])	1,895 [1,235, 2,467]
Etiology, *n* (%)	
ACM	1 (8%)
CTRCD	1 (8%)
HCM^a^	1 (8%)
DCM	6 (50%)
ICM	3 (25%)
Tissue originating procedure, *n* (%)	
Heart transplantation	10 (83%)
LVAD	1 (8%)
Postmortem	1 (8%)
Class 4/5 mutations, *n* (%)	
*LMNA*	1 (8%)
*PLN*	2 (17%)
*TTN*	3 (25%)
None	6 (50%)
Comorbidities, *n* (%)	
Diabetes mellitus	2 (17%)
Clinically relevant ventricular tachycardia	4 (33%)
Atrial fibrillation	3 (25%)
Medication, *n* (%)	
ACEi /ARB/ARNI	7 (58%)
Beta-blocker	3 (25%)
Calcium antagonist	6 (50%)
Class 3 antiarrhythmics (amiodarone)	8 (67%)
Glycoside	1 (8%)
MRA	6 (50%)
SGLT2-inhibitor	2 (17%)
# Slices, *n*	138

Abbreviations: ACEi, angiotensin-converting enzyme inhibitors; ACM, arrhythmogenic cardiomyopathy; ARB, angiotensin receptor blocker; ARNI, angiotensin receptor/neprilysin inhibitor; CTRCD, cancer therapy-related cardiac dysfunction; DCM, dilated cardiomyopathy; HCM, hypertrophic cardiomyopathy; ICM, ischemic cardiomyopathy; *PLN*, phospholamban; LVAD, left ventricular assist device; *LMNA*, lamin A/C; LMS, living myocardial slices; LVAD, left ventricular assist device; M, male; MRA, mineralocorticoid receptor antagonist; *TTN*, titin; SGLT2, sodium-glucose cotransporter 2.

a HCM in dilating phase.

**Table 2:** Cross-table Showing an Equal Distribution of Amiodarone Use and LVAD Implantation Across Etiologies

Etiology		Amiodarone (Y) (*N* = 7)	LVAD (Y) (*N* = 8)
Genetic	Non-*TTN* (*N* = 3)	2	2
	*TTN* (*N* = 3)	2	2
Nongenetic	Non-ICM (*N* = 3)	2	1
	ICM (*N* = 3)	1	3

Abbreviations: ICM, ischemic cardiomyopathy; LVAD, left ventricular assist device; *TTN, titin.*

### Simultaneous optical calcium transient and action potential assessment

To examine calcium transients, LMS were incubated for 15 minutes with 25 µM Rhod2AM (Invitrogen, Oregon) supplemented with 10 µM blebbistatin and 0.15% Pluronic F-127 (Sigma-Aldrich) in a modified Tyrode’s solution (1.8 mM CaCl_2_, 10 mM glucose, 10 mM HEPES, 4.5 mM KCl, 1 mM MgCl_2_, and 140 mM NaCl at pH 7.4). Subsequently, the LMS were incubated with 90 µM RH237 (Santa Cruz Biotechnology, Dallas, TX) and 10 µM blebbistatin for 15 minutes to visualize action potentials and de-esterification of the Rhod2AM. The LMS were then transferred to an in-house designed recording chamber, which was perfused with oxygenated modified Tyrode’s solution heated to 37 °C. A 10-ms biphasic pulse field stimulation was applied (Stimulator CS, Hugo Sachs Elektronik, Germany) with varying frequencies (0.5, 1, 2, 3 Hz, 30-second recordings of each). A custom-built microscope (Cairn Research, Faversham, UK) with a macroobjective (MVPLAPO 1X, OLYMPUS, Tokyo, Japan) was used to record the fluorescent signal. White light was filtered using a 545/30-nm excitation filter and directed onto the LMS with a 580-nm dichroic mirror. The resulting fluorescent signal was split using a 605/55-nm emission filter for Rhod2 and a 775/140-nm long-pass emission filter for RH 237. Images were captured by a high-speed camera (Andor Zyla 5.5 CL3, Oxford Instruments, Abingdon, UK). Data analysis was performed using Fiji and a custom-written MATLAB script, Peaks (doi:10.17605/OSF.IO/86UFE). The calcium amplitudes are reported relative to the diastolic levels.

### Extraction of amiodarone isolated from tissue

A modified liquid-liquid extraction was employed to isolate amiodarone from tissue, an adaptation to a previously published protocol.^6^ Tissue samples, including freshly generated LMS and corresponding epicardial fat from the same patient, as a positive control, were homogenized by bead-beating in ultrapure water at a ratio of 3 mL/g. From the homogenized mixture, 400 µL aliquots were spiked with 125 µL of an internal standard containing amiodarone-D4, prepared according to the manufacturer (MassTox antiarrhythmic drugs no. 92052, no. 92746, and quality control no. 0266, ChromSystems Instruments & Chemicals GmbH, Gräfelfing, Germany). Proteins were precipitated by adding acetonitrile in a 2:3 ratio. The mixture was vortexed and centrifuged at 25,000×*g* for 10 minutes at 4 °C. The organic supernatant was transferred to a glass tube, and 1 mL of n-hexane was added. After vortex and centrifugation at 25,000×*g* for 10 minutes at 4 °C, the upper organic layer was collected, repeating the extraction twice. The combined organic extracts were then evaporated until dry at 50 °C under N₂ vapor and the residue was reconstituted in methanol.

### Amiodarone detection and quantification

Amiodarone levels were quantified using a Vanquish UHPLC system coupled to a TSQ Quantum Access Max mass spectrometer (Thermo Fisher Scientific), following a routine clinical diagnostic protocol. A total of 15 µL of extracted samples, calibration standards, and quality control samples were injected for analysis. Chromatographic separation was performed using an Atlantis T3 column (2.1 × 100 mm, 3 µm, Waters, Milford, MA), maintained at 30 °C. The mobile phases used were 0.1% formic acid in water (mobile phase A) and 0.1% formic acid in acetonitrile (mobile phase B). The flow rate was set to 600 µL/min, and the total run time was 5 minutes. Linear gradient elution was used, starting with 5% mobile phase B, increasing to 98% at 2.5 minutes, maintained at 98% until 3.25 minutes, then returning to 5% at 3.251 minutes, and held constant until 5 minutes. All solvents were liquid chromatography-mass spectrometry (LC-MS) grade and obtained from Sigma-Aldrich. The mass spectrometer was operated in positive ion mode using selected reaction monitoring transitions for amiodarone (646.29-100.10 *m/z*) and its internal standard, amiodarone-D4 (649.95-104.15 *m/z*). The collision energy was set to 29 V, with a spray voltage of 3.5 kV. The ion transfer tube temperature was maintained at 270 °C, and the vaporizer temperature at 350 °C. Auxiliary and sheath gas pressures were set to 15 and 35 Arb, respectively. The area of the eluting peaks for both amiodarone and its D4 internal standard was determined. Liquid-liquid extraction recovery differences were corrected using the D4 internal standard, and the total amiodarone concentration was calculated accordingly.

### Statistical analysis

All statistical analyses were performed using R (v4.2.2) in R Studio and GraphPad Prism 10.2.2. Patient characteristics of the samples used to generate the LMS were summarized using the tableone package in R. Euclidean distances were calculated using the dist() function in R with the matrix of functional variables using the principal component analysis (PCA) as input. For comparisons between 2 groups, Welch's *t*-test was applied. For comparisons involving multiple groups, 1-way analysis of variance (ANOVA) was conducted, followed by posthoc analysis using Tukey's multiple comparisons method. In cases of repeated measurements, a mixed-effects approach was employed, again followed by posthoc analysis using Tukey's multiple comparisons method. All data are presented as mean ± standard error of the mean. A significance level of *p* < 0.05 was chosen and considered significant for all statistical tests.

## Results

### LMS patient variability and the influence of LVAD-induced mechanical unloading

**Figure 1:**
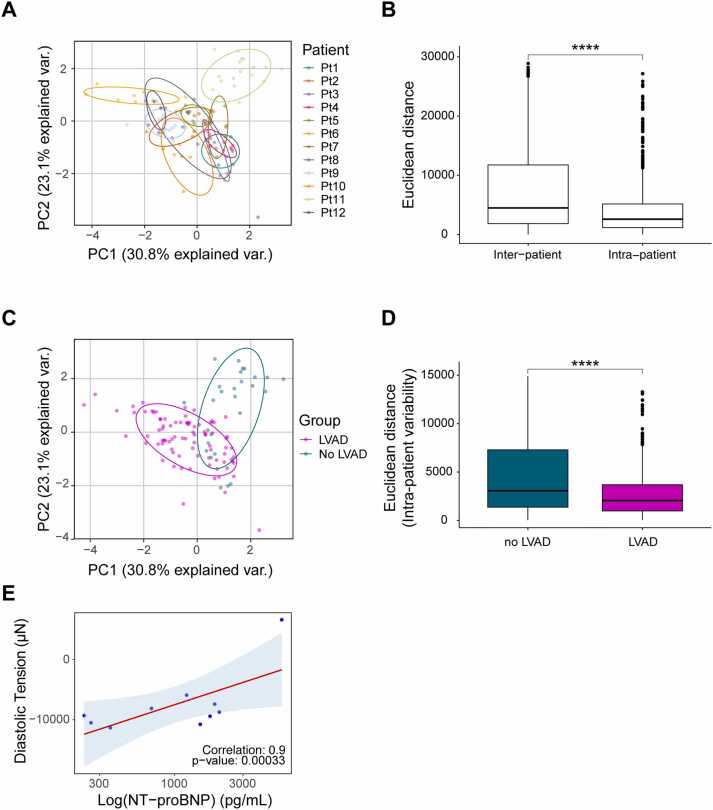
LMS reflect patient-specific functional variability and the impact of LVAD-induced mechanical unloading. (A) PCA of key functional parameters (refractory period, threshold potential, contractile force, post pause potentiation at 30 seconds, diastolic tension, and force-frequency relationship at 240 bpm), showing patient-specific grouping. Ellipses represent the standard deviation per patient. (B) Euclidean distance analysis reveals lower intrapatient functional variability compared to interpatient variability (A and B: *N* = 12, *n* = 138). (C) PCA was regrouped by the presence or absence of LVAD at the time of heart explantation and LMS preparation. (D) Euclidean distances between LVAD and no-LVAD patients highlight a decreased intrapatient variability in LVAD patients (C and D: LVAD, *N* = 8, *n* = 73; no LVAD, *N* = 2, *n* = 29). (E) Positive correlation between NT-proBNP levels, a marker for ventricular pressure and stress, and diastolic tension in LMS (E: *N* = 10, *n* = 102). A Wilcoxon rank-sum test was used to compare Euclidean distances, and Pearson correlation was performed to assess associations with NT-proBNP. *p* < 0.05 was considered statistically significant. LMS, living myocardial slices; NT-proBNP, N-terminal pro-B-type natriuretic peptide; LVAD, left ventricular assist device; PCA, principal component analysis.

HF is characterized by heterogeneity in its underlying cause, progression, and clinical presentation. The degree of tissue remodeling and accompanying impairment of cardiac function are highly variable. To determine whether patient-specific functional properties are retained in LMS, we assessed key functional parameters, including refractory period, threshold potential, contractile force, post pause potentiation at 30 seconds, diastolic tension, and force-frequency relationship at 240 bpm (*N* = 12, *n* = 138). PCA was used to summarize variance and reveal grouping patterns, with ellipses representing the functional standard deviation of each patient (Figure 1A). The contribution of each functional parameter to patient grouping was visualized using vector arrows, where arrow length indicates the strength of influence (Figure S1A). To quantitatively compare variability, Euclidean distances were calculated; this Euclidean distance is used as a proxy for functional similarity between pairwise slices in the space determined by the functional LMS variables included in the PCA. This analysis reveals lower intrapatient variability (slice-to-slice variability of the same patient) compared to interpatient variability (slice-to-slice variability between patients). This demonstrates the robust reproducibility of LMS and their ability to reflect the unique functional characteristics of the patient’s myocardial tissue (Figure 1B).

To assess how patient-specific factors, such as pre-existing LVAD-induced mechanical unloading, influence the tissue properties of LMS, we analyzed functional variability between tissues derived from patients with and without LVADs before heart transplantation (LVAD, *N* = 8, *n* = 73; no LVAD, *N* = 2, *n* = 29). Regrouping tissues based on LVAD status revealed distinct grouping patterns (Figure 1C), and Euclidean distance analysis confirmed reduced intrapatient variability in LVAD-treated patients, which could indicate a reduced transmural difference due to a reduced and equalized distribution of wall stress across the myocardium (Figure 1D). To confirm these findings, we analyzed the correlation with plasma N-terminal pro-B-type natriuretic peptide (NT-proBNP) levels before heart explanation, a continuous biomarker of disease severity and ventricular wall stress.^7^ NT-proBNP levels, which were all well above the clinical threshold for advanced HF (125 pg/ml), were stratified into high and low categories (cutoff: 1,700 pg/ml) as a surrogate for the differences in myocardial loading. This stratification revealed grouping patterns similar to those observed for LVAD and no-LVAD samples (Figure S1B). NT-proBNP levels significantly correlated with diastolic tension in LMS (Figure 1E), a parameter reflecting myocardial stiffness and potentially indicative of diastolic dysfunction, while other functional parameters did not reach statistical significance (Figure S1D-S1H). A similar trend in diastolic tension was observed between LVAD and no-LVAD groups (Figure S1C). Thus, LMS retained patient-specific functional properties and reflected characteristics associated with LVAD-induced mechanical unloading.

### Functional characterization of LMS based on etiology

To gain insights into the interpatient variability of LMS, we stratified the patients based on their HF etiology to investigate how the underlying cause influences myocardial functional characteristics. The onset of HF can be attributed to ischemic cardiomyopathy (ICM), caused by coronary artery disease and not a primary myocardial condition, or nonischemic cardiomyopathy (non-ICM), which is due to primary myocardial abnormalities and often has a genetic basis. Non-ICM was further stratified into titin (*TTN)* and non-*TTN* subgroups due to the high prevalence of *TTN* mutations and the associated better prognosis of these patients^8^, ^9^ (*TTN*, *N* = 3, *n* = 31; non-*TTN*, *N* = 3, *n* = 48; ICM, *N* = 3, *n* = 21; non-ICM, *N* = 3, *n* = 38).

**Figure 2:**
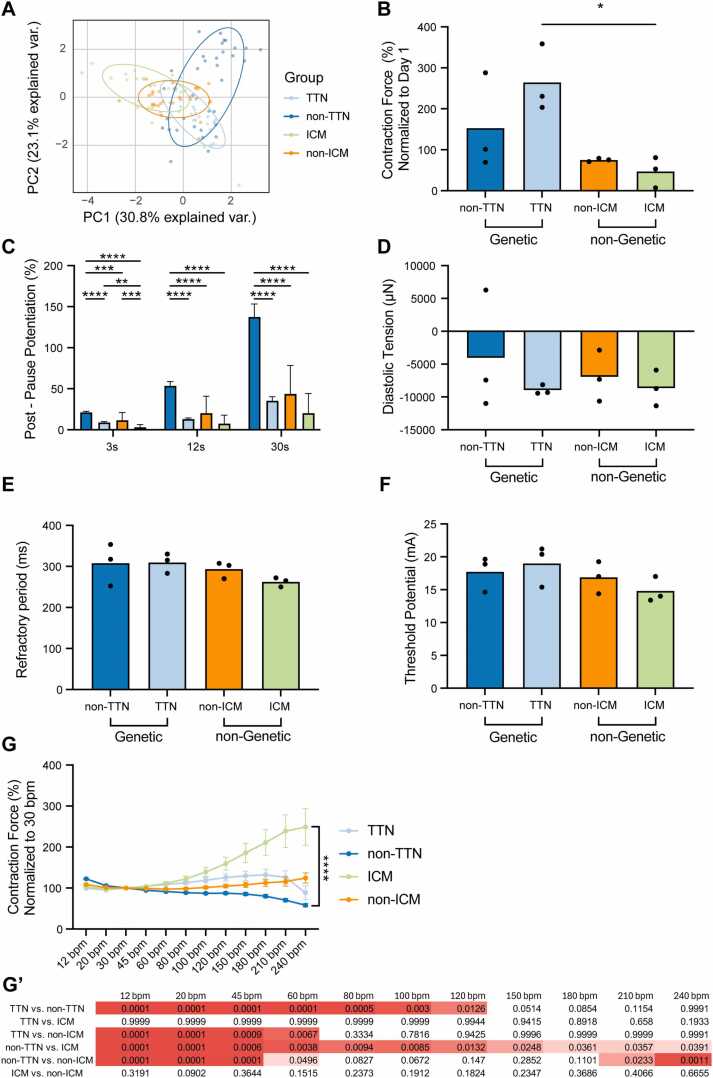
Functional characterization of LMS based on patient etiology. (A) PCA of key functional parameters showing distinct clustering of LMS based on etiology (*TTN*, non-*TTN*, ICM, and non-ICM). Ellipses represent the standard deviation per patient. (B) LMS from patients with a *TTN* mutation exhibit stronger contractile force compared to other etiologies. (C) Post pause potentiation after a 3-, 12-, and 30-second pause is significantly higher in LMS from non-*TTN* groups, particularly for longer pause durations. (D) Interaction between etiology and pacing frequency reveals distinct responses: LMS from non-*TTN* etiology show stronger contractile force at lower frequencies, while LMS from ICM patients demonstrate enhanced force at higher frequencies (A-D: *TTN*, *N* = 3, *n* = 31; non-*TTN*, *N* = 3, *n* = 48; ICM, *N* = 3, *n* = 21; non-ICM, *N* = 3, *n* = 38). One-way ANOVA with Tukey’s posthoc test and mixed-effects analysis were used where appropriate. *p* < 0.05 was considered statistically significant. ICM, ischemic cardiomyopathy; LMS, living myocardial slices; *TTN*, titin.

PCA of functional parameters revealed a distinct clustering of LMS samples based on their etiology (Figure 2A). LMS with a *TTN* mutation demonstrated the strongest contractile force (Figure 2B). Post pause potentiation, measured at 3, 12, and 30 seconds, was significantly higher in LMS derived from non-*TTN* groups compared to other etiologies, particularly for longer pause durations (Figure 2C). Other functional parameters, including diastolic tension, refractory period, and threshold potential, were comparable across etiologies (Figure 2D-F). LMS demonstrated a significant interaction between the originating etiology and its force-frequency relationship, indicating that the functional response of the myocardial tissue to changes in pacing frequency varies according to the underlying cause of HF. Specifically, LMS derived from patients with non-*TTN* etiology exhibited stronger contractile force at lower pacing frequencies, whereas LMS from ICM patients showed enhanced contractile force at higher pacing frequencies (Figure 2G and G’). These findings underscore how LMS capture patient-specific functional features that reflect the etiology of the diseased tissue.

### Effect of amiodarone on LMS functionality

**Figure 3:**
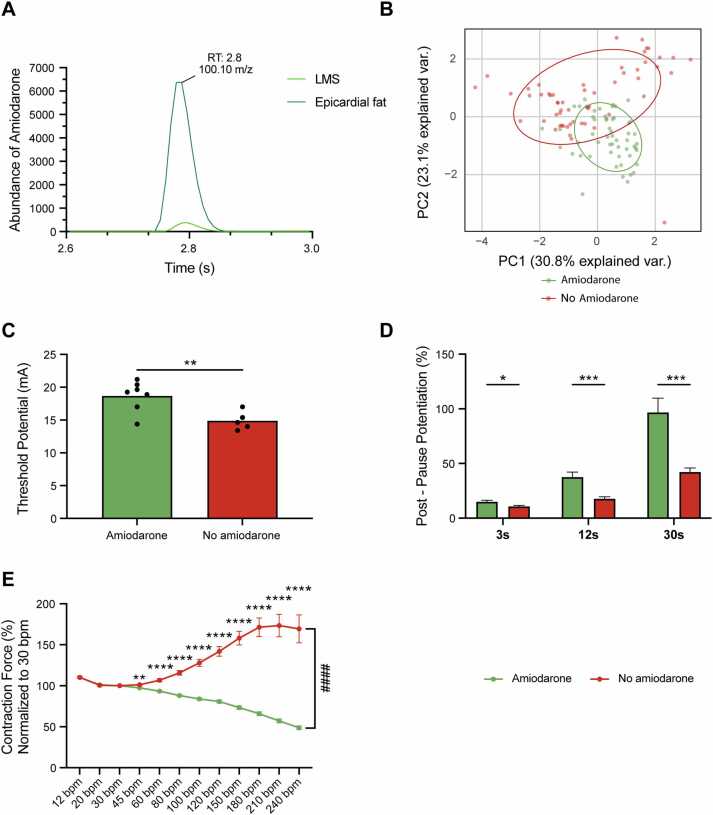
Effect of amiodarone on LMS function. (A) A representative LC-MS trace showing the retention time (RT) of 2.8 seconds, corresponding to amiodarone isolated from epicardial fat and LMS, as confirmed by comparison with the amiodarone standard. (B) PCA of functional parameters showing distinct clustering of LMS from amiodarone-treated vs untreated patients. (C) LMS from amiodarone-treated patients exhibit a higher threshold potential. (D) Post pause potentiation is enhanced in amiodarone-treated LMS, particularly at longer pause durations. (E) Force-frequency assessment reveals weaker contractile force in LMS from amiodarone-treated patients at higher pacing frequencies. (B-E: amiodarone *N* = 7, *n* = 66; no amiodarone *N* = 5, *n* = 70). Welch’s *t*-test and mixed-effects models with posthoc analysis were used where appropriate. *p* < 0.05 was considered statistically significant. LMS, living myocardial slices; PCA, principal component analysis.

To assess the impact of pharmacological regimens of the patient on LMS, we focused on amiodarone, a widely used antiarrhythmic agent known for its highly lipophilic nature^6^, ^10^ (amiodarone *N* = 7, *n* = 66; no amiodarone *N* = 5, *n* = 70). This property allows it to accumulate in tissues, reaching concentrations 10 to 50 times higher in the myocardium and 100 to 1,000 times higher in adipose tissue compared to plasma levels.^10^ Using a modified liquid-liquid extraction,^6^ amiodarone was successfully extracted from LMS and epicardial tissue, with levels in epicardial fat ranging from 267.6 to 759.9 μg/g and in LMS ranging from 8.6 to 38.1 μg/g, representing a 10- to 30-fold difference (Figure S2A-C). A representative LC-MS trace of amiodarone levels in epicardial fat and LMS from the same patient is shown in Figure 3A. PCA demonstrated a distinct functional profile of LMS derived from amiodarone-treated and untreated patients (Figure 3B). LMS from amiodarone-treated patients exhibited a higher threshold current (Figure 3C) and enhanced post pause potentiation, particularly for longer pauses (Figure 3D). Additionally, LMS from treated patients generated stronger contractile force at higher pacing frequencies, demonstrating a positive force-frequency relationship (Figure 3E). No statistically significant differences were observed in the refractory period, diastolic tension, and contractile force (Figure S2D-F).

### Effect of amiodarone on LMS electrophysiological properties

**Figure 4:**
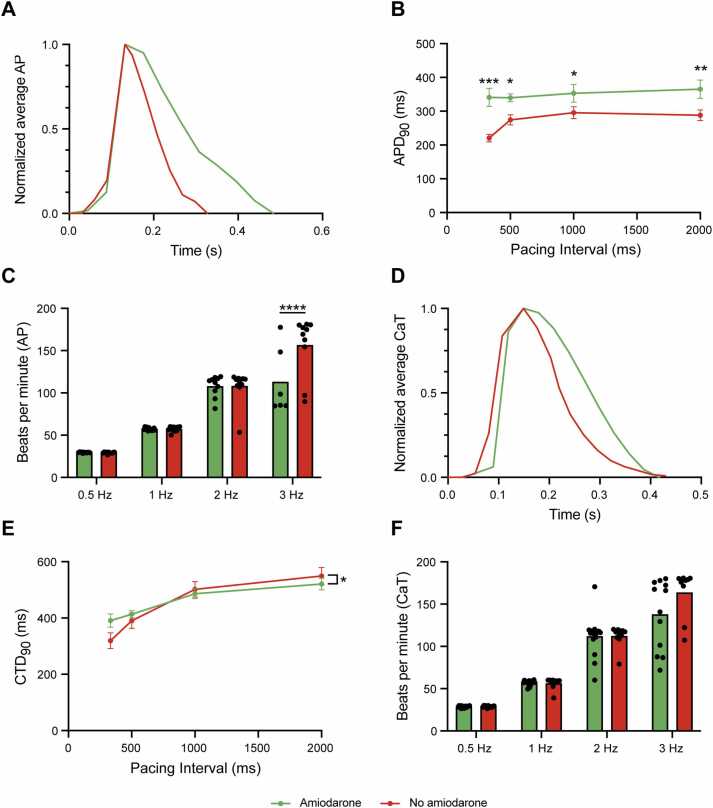
Effect of amiodarone on LMS electrophysiological properties. (A) LMS from amiodarone-treated patients exhibit prolonged APD_90_ compared to untreated LMS. (B) APD_90_ in treated LMS fails to adapt to increased pacing frequency. (C) LMS derived from amiodarone-treated patients are unable to maintain pacing at 3 Hz, unlike the LMS from patients without amiodarone. (D, E) CTD_90_ shows an interaction between pacing interval and amiodarone treatment, though no significant differences are observed in posthoc analysis. (F) Calcium transient analysis reveals an inability of LMS from patients treated with amiodarone to follow pacing at 3 Hz, though less pronounced than for action potentials. (A-F: amiodarone, *N* = 6, *n* = 17; no amiodarone, *N* = 4, *n* = 11). A mixed-effects model with posthoc analysis was used to compare amiodarone and no amiodarone. *p* < 0.05 was considered statistically significant. APD, action potential duration; CTD, calcium transient duration; LMS, living myocardial slices.

Amiodarone is well-known for its effect on prolonging action potential duration (APD) and altering calcium handling.^11^ To evaluate these effects on LMS, we performed simultaneous optical assessments of action potential and calcium dynamics (amiodarone, *N* = 6, *n* = 17; no amiodarone, *N* = 4, *n* = 11). LMS derived from amiodarone-treated patients exhibited a prolonged APD_90_ compared to untreated samples, with an inability to adapt APD to increased pacing frequency (Figure 4A and B). Additionally, LMS from amiodarone-treated patients were unable to follow a pacing of 3 Hz, unlike their respective untreated counterparts (Figure 4C).

Calcium transient duration (CTD_90_) showed a significant interaction between pacing interval and amiodarone treatment, although posthoc analysis did not show significant differences between groups (Figure 4D and E). Similarly, calcium transient assessment confirmed a reduced ability of amiodarone-treated LMS to follow pacing at 3 Hz, though the effect was less pronounced than for action potentials (Figure 4F). Calcium transient amplitude remained unchanged between groups (Figure S2G).

Interestingly, LMS derived from amiodarone-treated patients exhibit a comparable frequency dependency (slope, *p* = 0.15) for the difference between CTD_90_ and APD_90_, but the difference is consistently smaller compared to LMS derived from patients not using amiodarone (intercept, *p* = <0.001). This difference, a measure of excitation-contraction coupling, reflects that amiodarone prolongs APD_90_ while minimally affecting CTD_90_ (Figure S2H). These findings align with amiodarone’s known mechanism of modulating excitation-contraction coupling, stabilizing both electrical and mechanical activity. While these effects contribute to its antiarrhythmic properties and myocardial contractile stability, they also limit the responsiveness to high-frequency pacing.

## Discussion

### LMS as a translational model by retaining patient-specific functional features

HF is marked by complex, patient-specific variability that hinders effective treatment. Addressing this challenge requires translational models that bridge the gap between experimental findings and clinical applications. LMS are an increasingly used translational platform that preserve native cardiac structure and multicellularity and can be generated from both animal and human myocardium. Although originally introduced in the mid-20th century,^12^ LMS have gained renewed attention in recent years due to advances in biomimetic culture systems that enable prolonged viability and functional assessment.^13^, ^14^, ^15^ Their preparation requires access to fresh cardiac tissue, which in the human setting, depends on a close interdisciplinary collaboration between cardiologists, surgeons, and experimental teams. The technique relies on a high-precision vibratome, and generating and handling slices has a steep technical learning curve. Nevertheless, the growing availability of open-access protocols^16^ and efforts to standardize methodologies^17^ are making the approach increasingly accessible. However, to this day, it is not clear to what extent LMS retain the characteristics of the patients from whom they were derived.

By analyzing intrapatient and interpatient variability, we show that LMS retain patient-specific functional features, by displaying a lower intrapatient compared to interpatient variability. In our study on a diverse end-stage HF patient population, LMS demonstrated functional characteristics consistent with their etiology, amiodarone usage, and mechanical unloading.

These findings establish LMS as a robust translational platform for investigating the functional impact of clinical conditions and therapeutic interventions. Moreover, this validates the potential role of LMS in personalized medicine, as illustrated by prior case reports employing LMS.^18^, ^19^

### The impact of mechanical load on LMS function

LMS from patients without prior LVAD implantation exhibited a greater intrapatient variability and showed a higher diastolic tension, which correlated with NT-proBNP plasma levels. NT-proBNP levels are known to decrease after LVAD implantation as mechanical unloading alleviates stress and pressure on the ventricular wall.^20^ Mechanical unloading of a failing heart through LVAD therapy may induce reverse remodeling, a process that restores cardiac structure, reduces fibrosis, and normalizes stiffness.^21^, ^22^, ^23^ In LMS, this remodeled state is characterized by a reduction in diastolic tension, which improves myocardial relaxation. The observed reduction in intrapatient variability and increased uniformity in myocardial properties in LVAD-treated patients is consistent with the literature, which indicates that mechanical unloading reduces transmural differences, likely by promoting more uniform remodeling and restoring myocardial wall thickness.^24^ While this study employs a cross-sectional design, future longitudinal sampling, for example, at LVAD implantation and again at the time of transplantation, could better capture dynamic myocardial adaptations to mechanical circulatory support and contribute to a clearer understanding of recovery trajectories, which remain poorly defined.

### The impact of etiology on LMS function

The distinct functional profiles observed in LMS derived from various etiologies reflect the model's capacity to capture patient-specific cardiac properties. LMS with non-*TTN* genetic cardiomyopathies, consisting of mutations in *PLN* and *LMNA* genes, exhibited elevated post pause potentiation and efficient contractile force at low pacing frequency. These effects are consistent with their known disease mechanisms involving disrupted calcium handling and excitation-contraction coupling, contributing to arrhythmogenic phenotypes through sarcoplasmic reticulum calcium overload, delayed after depolarizations, and electrical instability.^25^, ^26^, ^27^, ^28^, ^29^, ^30^ LMS from patients with *TTN* mutations exhibited the least impaired contractile force, aligning with studies indicating that such mutations have a better prognosis and may result in adaptive changes of *TTN*’s elastic properties, potentially contributing to preserved contraction force.^8^, ^9^, ^31^, ^32^ Conversely, a physiological positive force-frequency relationship was exclusively observed in LMS from ICM patients. ICM exhibits a nonuniform pattern in the heart, primarily affecting the infarct zone and peri-infarct regions; selecting myocardial tissue distant from these ischemic regions for biopsy of LMS generation could account for this retained physiological response. Altogether, these results demonstrate that LMS retain the functional effects of different pathophysiological mechanisms, offering valuable insights into the functional consequences of specific cardiac diseases and potential therapeutic interventions.

### The impact of pharmacological intervention on LMS function

This study is the first to demonstrate that LMS retain clinical patient-specific features, extending even to the effects of pharmacological interventions *in vivo*. LMS from amiodarone-treated patients exhibited prolonged APD, consistent with the clinical effect of amiodarone to inhibit sodium, potassium, and calcium channels.^11^ Enhanced post pause potentiation, reduced excitability, and a flattened force-frequency relationship further underscore changes in sarcoplasmic reticulum calcium dynamics and excitation-contraction coupling, aligning with the drug’s antiarrhythmic and stabilizing effects on cardiac electrical activity and contraction dynamics.^33^ LMS from amiodarone-treated patients also demonstrated impaired electrical adaptability, including difficulty following pacing at higher frequencies, in line with amiodarone’s pharmacological effects.^34^

These effects appear specific to amiodarone rather than intrinsic patient characteristics, as the functional outcomes align with its known pharmacological effects. Furthermore, the balanced distribution of patient subgroups shown in Table 2 further minimizes the risk that clustering or functional differences are driven by clinical confounders rather than pharmacological effects. Notably, adding amiodarone directly to LMS *ex vivo* mimics only its acute ion channel effects, as it lacks the sustained *in vivo* drug accumulation and integration into myocardial tissue dynamics, partly due to adsorption by culture surfaces.^10^, ^35^ This emphasizes the ability of LMS to preserve pharmacologically induced modifications achieved through long-term *in vivo* exposure.

While amiodarone served as a proof-of-concept example in this study, LMS could similarly be used to investigate the mechanisms of other drugs that accumulate in myocardial tissue. These include but are not limited to beta-blockers (e.g., metoprolol^36^, ^37^), cardiac glycosides (e.g., digoxin^38^), as well as emerging therapeutic agents. Moreover, LMS offer a unique platform to study drug metabolism directly in the heart, bypassing systemic influences and providing insights into cardiac-specific metabolic pathways. The ability to recapitulate patient-specific pharmacological responses positions LMS as a valuable translational platform for studying therapeutic interventions and optimizing drug efficacy.

### Next steps in translational research

This study is the first to thoroughly and successfully translate LMS findings to patient-specific clinical characteristics, demonstrating their potential as a personalized translational model. By reflecting patient-specific functional characteristics, LMS have proven their utility as a translational model for exploring myocardial functionality, drug effects, and etiology-based variability. However, to fully achieve their translational potential, it is essential to maintain the highest quality in LMS preparation and experimental setup to reliably replicate patient-specific features.

Despite their promises, LMS face challenges stemming from the inherent variability in patient characteristics, such as underlying etiology, and pharmacological regimens, which lie beyond experimental control and must be carefully considered when designing studies. This variability complicates study design and highlights the need for large sample sizes. Accumulating LMS from patients with genetic mutations or specific conditions remains particularly challenging. Addressing these issues will require multicenter collaborations to pool resources, advanced computational methods such as machine learning to analyze small, heterogeneous datasets, and standardized protocols for LMS preparation and functional testing to ensure reproducibility and comparability. Alternatively, stable preservation of LMS or cardiac biopsies may enable the accumulation of specific patient samples.^39^, ^40^, ^41^

Future research should focus on expanding LMS applications by studying a broader array of pharmacological agents, exploring a wider range of etiologies, and strengthening correlations between LMS findings and *in vivo* clinical outcomes. These efforts will further establish LMS as a robust platform for personalized cardiac research, advancing our understanding of cardiac diseases and optimizing patient-specific therapeutic interventions.

## Conclusion

In conclusion, this study demonstrates the potential of LMS as a translational model for personalized cardiac research. By retaining patient-specific functional characteristics, LMS provide a unique platform to bridge the gap between experimental models and clinical applications. As we expand the scope of LMS studies to include broader pharmacological investigations, diverse etiologies, and *in vivo* validations, this platform holds the promise of revolutionizing our understanding of cardiac diseases and advancing precision medicine for improved patient outcomes.

## CRediT authorship contribution statement

**Jort S.A. van der Geest:** Conceptualization, Data curation, Formal analysis, Investigation, Methodology, Project administration, Resources, Validation, Visualization, Writing – original draft, Writing – review & editing. **Willem B. van Ham:** Data curation, Formal analysis, Writing – review & editing. **Ernest Diez Benavente:** Methodology, Supervision, Visualization, Writing – review & editing. **Mohsin El Amrani:** Methodology, Writing – review & editing. **M. Mostafa Mokhles:** Resources, Writing – review & editing. **Marish I.F.J. Oerlemans:** Resources, Writing – review & editing. **Pieter A. Doevendans:** Funding acquisition, Supervision, Writing – review & editing. **Teun P. de Boer:** Resources, Supervision, Writing – review & editing. **Joost P.G. Sluijter:** Funding acquisition, Supervision, Writing – review & editing. **Linda W. van Laake:** Resources, Supervision, Writing – review & editing. **Vasco Sampaio-Pinto:** Conceptualization, Data curation, Formal analysis, Investigation, Methodology, Project administration, Supervision, Validation, Writing – review & editing.

## Disclosure statement

The authors declare the following financial interests/personal relationships which may be considered as potential competing interests: Joost P.G. Sluijter reports that financial support was provided by Netherlands Organisation for Health Research and Development, Dutch Research Council, and European Research Council. Linda W. van Laake reports that financial support was provided by Netherlands Heart Foundation. Ernest Diez Benavente reports that financial support was provided by European Research Council. Pieter A. Doevendans reports that financial support was provided by Horizon Europe. Vasco Sampaio-Pinto reports that financial support was provided by Netherlands Heart Institute. The other authors declare that they have no known competing financial interests or personal relationships that could have appeared to influence the work reported in this paper.

We are grateful for the close collaboration between cardiothoracic surgeons (CTC), cardiologists, the OR team, heart failure nurses, and VAD coordinators at UMC Utrecht in procuring fresh cardiac tissue.

J.S.A.vd.G. P.A.D. and J.P.G.S. are supported by ZonMw Psider-Heart (10250022110004), NWO-TTP HARVEY (2021/TTW/01038252), the PLN Foundation and ERA for Health Cardinnov (RESCUE- 2024/KIC/01627794). Additionally J.P.G.S. is supported by H2020-EVICARE (725229) of the 10.13039/501100000781European Research Council (ERC); L.W.v.L. is supported by the 10.13039/501100002996Dutch Heart Foundation: Dekker Senior Clinical Scientist 2019, grant agreement number 2019T056 and by the Alliance Fund (UMCU, UU, TU/e); E.D.B. is funded by the European Union project European Research Council consolidator grant 866478 (UCARE) and the Leducq Foundation AtheroGEN; P.A.D. is supported by CUREPLaN Leducq (18CVD01), and EU Horizon 2022 grant GEREMY (101080204). V.S.-P. is supported by a 10.13039/501100014470Netherlands Heart Institute postdoctoral fellowship.

## Appendix A Supplementary data

**Supplemental Figure 1** (A) The vector arrows underlying the PCA of key functional parameters indicate the contribution of each parameter. (B) PCA grouped by NT-proBNP levels (high vs low, with a threshold of 1,700 pg/mL) recapitulates clustering patterns comparable to those observed in LVAD and no-LVAD samples (high *N* = 4, *n* = 43; low *N* = 6, *n* = 54). (C) LMS from LVAD patients show a decreased trend for diastolic tension compared to non-LVAD patients, in line with NT-proBNP results (LVAD *N* = 8, *n* = 70; no LVAD *N* = 2, *n* = 27). Linear correlation of NT-proBNP levels with key functional parameters, including contractile force at 240 bpm (D), contraction force normalized to day 1 (E), refractory period (F), stimulation threshold (G), and 30-second post pause potentiation (H) (D-H: *N* = 10, *n* = 102). Welch’s *t*-test was used for 2-group comparisons; Pearson's correlation was used to assess associations with NT-proBNP. *p* < 0.05 was considered statistically significant

.

**Supplemental Figure 2** Raw LC-MS quantifications of amiodarone extracted from epicardial fat and LMS, showing the area under the curve (AUC) (A), recovery of the internal standard amiodarone-D4 (B), and the corrected amiodarone quantification per gram of epicardial fat and myocardial tissue (C). LMS from amiodarone-treated patients exhibit no difference in refractory period (D), diastolic tension (E), contraction force (F), and calcium amplitude (G) compared to untreated LMS. H) The ΔCTD90-APD90 as a function of pacing interval reveals a consistently smaller difference (*p* < 0.001) in amiodarone-treated LMS compared to untreated LMS, while the frequency dependency was comparable (*p* = 0.15) (E-H: amiodarone, *N* = 7, *n* = 66; no amiodarone, *N* = 5, *n* = 70). Welch’s *t*-test was used for comparisons between treatment groups. Linear regression was performed per group, and analysis of covariance (ANCOVA) was used to compare intercepts and slopes. *p* < 0.05 was considered statistically significant

## Footnotes

Appendix A Supplementary data associated with this article can be found in the online version at doi:10.1016/j.jhlto.2025.100345.
