# Supplementary figures and images for: Living myocardial slices retain patient-specific features: Insights into etiology and therapeutic history
Source: JHLT Open. 2025 Jul 14;9:100345. doi: 10.1016/j.jhlto.2025.100345 (PMC12341583; doi:10.1016/j.jhlto.2025.100345)

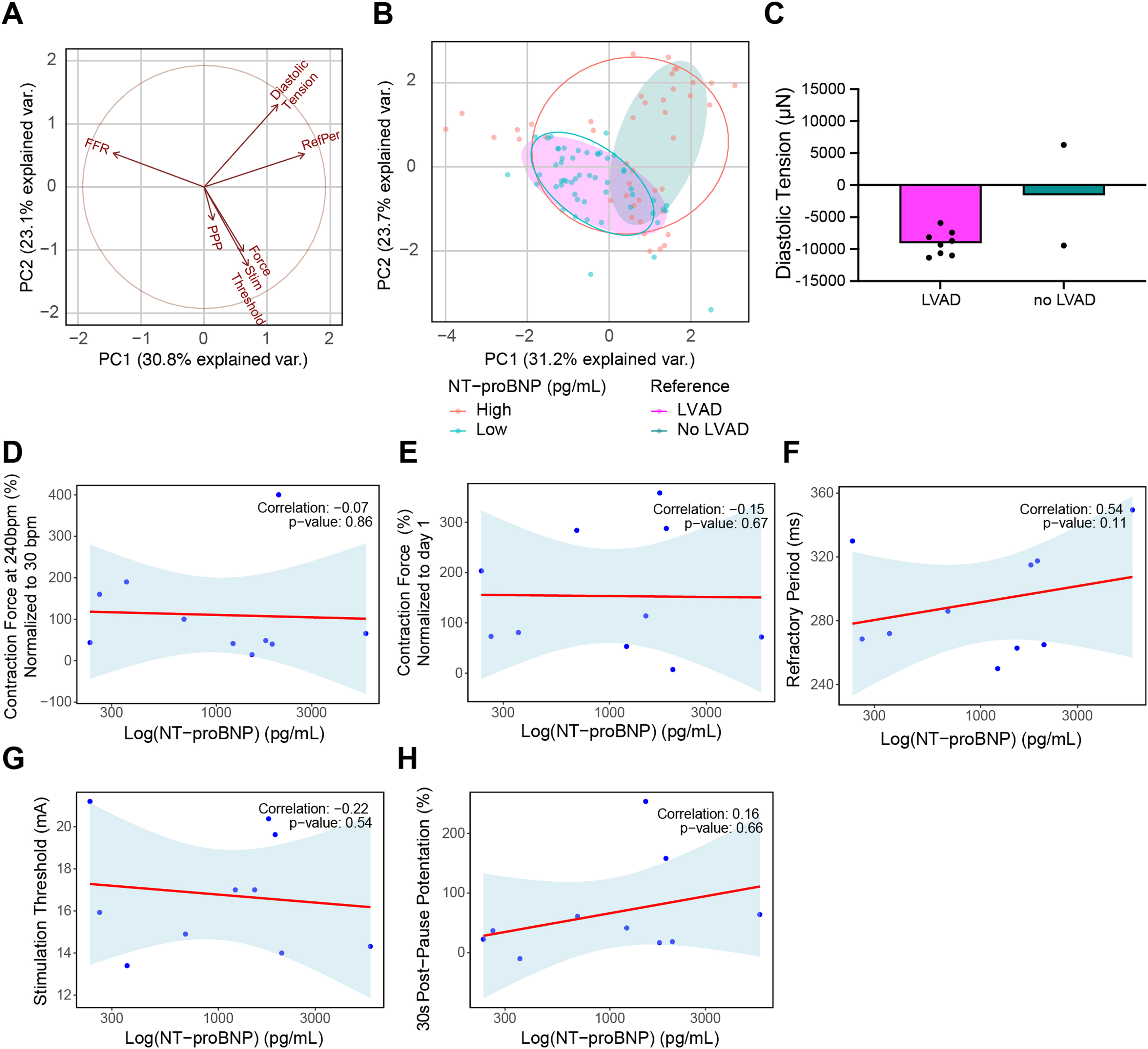

Supplement: Supplementary file 1 — Supplemental Figure 1 (A) The vector arrows underlying the PCA of key functional parameters indicate the contribution of each parameter. (B) PCA grouped by NT-proBNP levels (high vs low, with a threshold of 1,700 pg/mL) recapitulates clustering patterns comparable to those observed in LVAD and no-LVAD samples (high N = 4, n = 43; low N = 6, n = 54). (C) LMS from LVAD patients show a decreased trend for diastolic tension compared to non-LVAD patients, in line with NT-proBNP results (LVAD N = 8, n = 70; no LVAD N = 2, n = 27). Linear correlation of NT-proBNP levels with key functional parameters, including contractile force at 240 bpm (D), contraction force normalized to day 1 (E), refractory period (F), stimulation threshold (G), and 30-second post pause potentiation (H) (D-H: N = 10, n = 102). Welch’s t-test was used for 2-group comparisons; Pearson's correlation was used to assess associations with NT-proBNP. p < 0.05 was considered statistically significant [file mmc1.jpg]

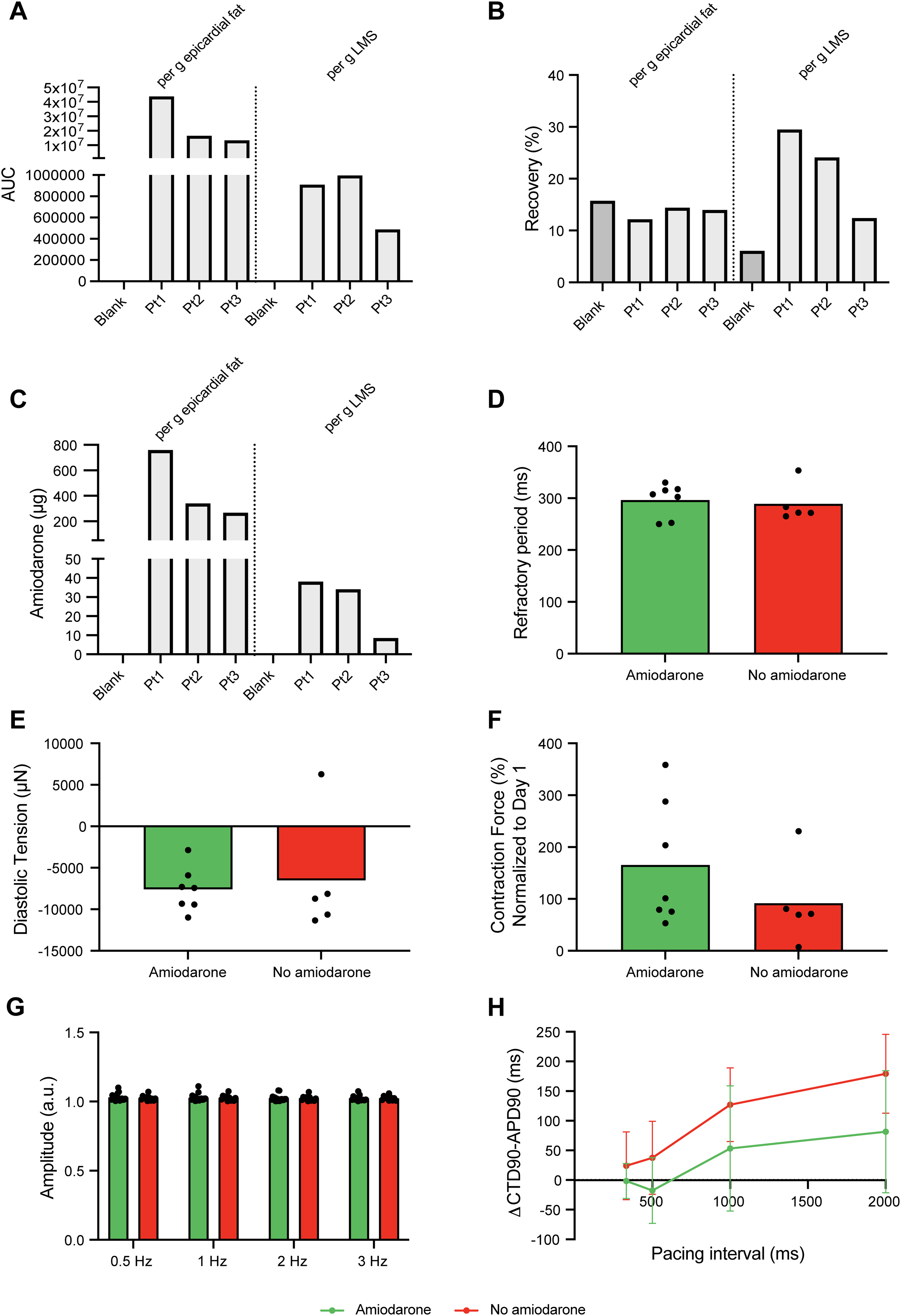

Supplement: Supplementary file 2 — Supplemental Figure 2 Raw LC-MS quantifications of amiodarone extracted from epicardial fat and LMS, showing the area under the curve (AUC) (A), recovery of the internal standard amiodarone-D4 (B), and the corrected amiodarone quantification per gram of epicardial fat and myocardial tissue (C). LMS from amiodarone-treated patients exhibit no difference in refractory period (D), diastolic tension (E), contraction force (F), and calcium amplitude (G) compared to untreated LMS. H) The ΔCTD90-APD90 as a function of pacing interval reveals a consistently smaller difference (p < 0.001) in amiodarone-treated LMS compared to untreated LMS, while the frequency dependency was comparable (p = 0.15) (E-H: amiodarone, N = 7, n = 66; no amiodarone, N = 5, n = 70). Welch’s t-test was used for comparisons between treatment groups. Linear regression was performed per group, and analysis of covariance (ANCOVA) was used to compare intercepts and slopes. p < 0.05 was considered statistically significant [file mmc2.jpg]
